# Behavioral profile of adults with Prader-Willi syndrome: correlations with individual and environmental variables

**DOI:** 10.1186/1866-1955-5-18

**Published:** 2013-08-06

**Authors:** Joseba Jauregi, Virginie Laurier, Pierre Copet, Maithé Tauber, Denise Thuilleaux

**Affiliations:** 1 Psikologia fakultatea, EHU- University of the Basque Country, Donostia, Spain; 2 Centre de Référence Prader-Willi, Hôpital Marin AP-HP, Hendaye, France

**Keywords:** Prader–Willi syndrome, Behavior, Genotype, Adults

## Abstract

**Background:**

Maladaptive behavior has been reported as a phenotypical feature in Prader–Willi syndrome (PWS). It severely limits social adaptation and the quality of life of children and adults with the syndrome. Different factors have been linked with the intensity and form of these behavioral disturbances but there is no consensus about the cause. Consequently, there is still controversy regarding management strategies and there is a need for new data.

**Methods:**

The behavior of 100 adults with PWS attending a dedicated center was assessed using the Developmental Behavior Checklist for Adults (DBC-A) and the PWS-specific Hyperphagia Questionnaire. The DBC-A was completed separately by trained caregivers at the center and relatives or caregivers in a natural setting. Genotype, gender, age, degree of obesity and cognitive impairment were analyzed as variables with a hypothetical influence on behavioral features.

**Results:**

Patients showed a relatively high rate of behavioral disturbances other than hyperphagia. Disruptive and social relating were the highest scoring DBC-A subscales whereas anxiety/antisocial and self-absorbed were the lowest. When hospital caregiver and natural caregiver scores were compared, scores for the latter were higher for all subscales except for disruptive and anxiety/antisocial. These effects of institutional management were underlined. In the DBC-A, 22 items have descriptive indications of PWS behavior and were used for further comparisons and correlation analysis. In contrast to previous reports, rates of disturbed behavior were lower in patients with a deletion genotype. However, the behavioral profile was similar for both genotypes. No differences were found in any measurement when comparing type I and type II deletions. The other analyzed variables showed little relevance.

**Conclusions:**

Significant rates of behavioral disorders were highlighted and their typology described in a large cohort of adults with PWS. The deletion genotype was related to a lower severity of symptoms. Some major behavioral problems, such as hyperphagia, may be well controlled if living circumstances are adapted to the specific requirements of individuals with PWS.

## Background

Several studies over the last few years have focused on behavioral aspects of genetic diseases, including Prader–Willi syndrome (PWS). There are at least two reasons for this. First, behavioral disturbances are often the most challenging expression of the syndrome and their assessment is the first step in developing efficient management strategies to improve the social adaptation and quality of life of patients and their families. Second, some behavioral characteristics are specific to these syndromes and shape a behavioral phenotype with a characteristic pattern of motor, cognitive, linguistic and social abnormalities, which are consistently associated with a genetic disorder. In this case, studying the behavior and underlying cognitive dysfunctions may increase our understanding of the genetic influence on normal and pathological behaviors in the general population. As a natural experiment, genetic syndromes bridge the gaps between genes, brain, cognition and behavior.

The methodology for assessing behavior in genetic syndromes with associated intellectual disability is controversial [1]. Because they are rare diseases, obtaining samples of an adequate size is difficult and the validation of specific questionnaires is often impossible. Recently, there have been proposals to improve the measurement of behavior in behavioral phenotype research [2]. In accordance with these, this study aimed to describe the behavioral profile of a large cohort of adults with PWS and to analyze the within-syndrome variability in relation to differences in genotype, age, gender, intellectual abilities and body mass index (BMI) using standardized measuring instruments. Also, we examined the influence of environment on behavioral features.

PWS (OMIM, 176270) is a developmental and multisystem genetic disorder characterized by typical dysmorphic features and a variable expression of endocrine, neurological, cognitive and behavioral symptoms [3,4]. It is a ‘contiguous gene syndrome’, which results from the absence of expression of paternally derived alleles of maternally imprinted genes in the q11-13 region of chromosome 15. In 70% of patients, the cause is a paternal deletion of 15q11-q13, and maternal uniparental disomy (m-UPD) is found in 25%. Other mechanisms are involved in the remaining 5% [5]. The deletion can be long (type I) or short (type II) [6]. The PWS genetic region contains a cluster of imprinted and non-imprinted genes for which the respective contributions to phenotypic features have not yet been clearly established [7].

It has been previously reported that people with PWS show higher rates of maladaptive behaviors than the general population and people with similar intellectual disability due to other etiologies [8-15]. Besides the typical hyperphagia, challenging behaviors commonly described among people with PWS include stubbornness, temper tantrums, skin picking, compulsivity, mood fluctuations and disruptive behavior [16-18]. Most of these studies were based on inventories for typically developing people or unvalidated checklists and focused on children or adolescents. The results are not always in agreement. Much less is known about behavioral difficulties in adults. Persistence of the same problems to different degrees has been reported [9,19,20] and it is also true that the severity and typology of behavioral problems can vary considerably across individuals with PWS.

There is no consensus on the influence of variables such as age, gender, BMI or intellectual disability level on PWS behavior. The relation between age and maladaptive behaviors has been described as non-linear, increasing as children get older [20,21] and with young adults showing the greatest incidence of problems. However, fewer maladaptive behaviors are exhibited by older adults than younger adults [20]. Some authors reported that males have more externalizing behaviors, aggression, and depression [16,20,22]. However, other studies did not find these gender differences [10,17,23]. The degree of obesity has been related to behavioral disturbances in contradictory ways. Some authors found no relationship [13,24,25], others found higher levels of maladaptive behavior in those with a lower BMI [16,20,22] whereas others found an aggravation of behavioral symptoms in those with a higher BMI [26]. Cognitive impairment, usually at a mild or moderate level [27,28], has been reported as not being associated with behavioral difficulties [24,25,29]. Cognitive and behavioral disorders in PWS have been related to frontal lobe dysfunctions [30,31].

It is becoming increasingly clear that relevant phenotypical differences exist between the two main genotypes in PWS: deletion and m-UPD. These differences are of somatic features [32], cognitive profiles [27,28] and psychiatric disorders [33,34]. Behavioral disturbances have also been reported to have a different typology or severity as a function of genotype. Previous studies suggested that the m-UPD subtype has milder maladaptive behaviors than the typical deletion subtype. Patients may be less apt to skin-picking [23,35], stealing food [36], hoarding and overeating [35] and display fewer obsessive-compulsive and ritualistic behaviors [29,37]. On the other hand, they have been reported as having a higher risk for autism spectrum disorders [38] and psychosis [39].

Behavioral differences between type I and type II deletions have also been reported but not always replicated. The proposed differences are for cognitive, adaptive and psychopathological issues [22,40,41]. However, other reports failed to find such differences [29,37,42].

A recent article [14] showed that for a Dutch cohort, there were higher levels of behavioral disorders in people with m-UPD compared with people with the deletion, and with the type I deletion compared with type II. This report is particularly interesting because it uses the same behavioral assessment instrument as us for a cohort similar to ours.

Taking into account the lack of agreement on behavioral issues of PWS, large descriptive cohort studies remain pertinent. In fact, little is currently known about the variables associated with the prevalence and severity of behavioral disturbances in individuals with PWS. This research aimed to increase knowledge of PWS by focusing on the behavioral aspects that often have significant impacts on affected individuals and their families. Furthermore, the effects of life settings specifically designed for patients with PWS were analyzed through a comparison between behavioral measurements in our unit and in their family homes.

## Methods

### Subjects

This study is based on the total population that was admitted at least once between July 2008 and the end of 2009 to a PWS-dedicated unit belonging to the French Reference Centre for PWS, which uses a multidisciplinary approach to the syndrome. Admissions were requested by the patient or his or her caregivers for a period of one to three months. The purpose of a stay is to assess the patient’s psychosocial and medical problems in order to define that person’s needs and to propose a personalized management strategy. The most frequent objectives were to control weight, to improve physical conditions, to treat medical complications and to promote psychological well-being and social interactions. Furthermore, a stay is a break from the family and everyday residential routines. Admissions were scheduled in advance, not in response to an aggravated clinical situation.

The cohort was thus composed of 100 adult individuals: 44 males and 56 females (Table 1). The age range of the subjects was 18 to 53 years (mean = 28.2; SD = 7.7). BMI ranged from 19 to 75 (mean = 42; SD= 10.6). All patients had a genetic confirmation of the diagnosis. Of the patients, 73 had a 15q11-q13 paternal deletion and comprised the deletion group (DEL). Ten patients had a confirmed maternal disomy and 13 patients had an abnormal methylation profile with negative results for the deletion diagnosis (Fluorescence In Situ Hybridation). We combined this group with the confirmed m-UPD group, assuming that the error risk would be acceptable (non-deletion and non-UPD cases comprised less than 5%). This combined group was called the non-deletion group (Non-DEL) and had 23 patients. Four other forms of genetic error were present (two imprinting center mutations and two translocations). A determination of the size of the deletion was available for 36 subjects: 10 had the longer form of deletion (type I, TI = BP1-BP3) and 26 had the shorter form (type II, TII = BP2-BP3).

**Table 1:** Patient characteristics

	**Whole group**	**Deletion group**	**Non-deletion group**	**Others**
Number of patients	100	73	23	4
Male	44	33	8	3
Female	56	40	15	1
Mean age (SD)	28.2 (7.7)	27.9 (7.6)	29.1 (8.2)	27.0 (6.1)
Mean BMI (SD)	42.0 (10.6)	44.4 (10.7)	35.9 (7.7)	35.0 (7.9)
Mean FSIQ (*n*, SD)	54.5 (84, 9.6)	55.5 (63, 10.2)	51.7 (18, 7.2)	51.7 (3, 2.1)
Mean VIQ (*n*, SD)	57.1 (84, 11.3)	57.4 (63, 12.2)	56.3 (18, 9.0)	54.0 (3, 1.7)
Mean PIQ (*n*, SD)	56.0 (84, 9.8)	57.8 (63, 10.3)	50.3 (18, 5.7)	53.7 (3, 5.0)

*BMI* body mass index, *FSIQ* full-scale IQ, *IQ* intelligence quotient, *PIQ* performance IQ, *VIQ* verbal IQ.

### Procedure

Most of the data for this study were collected during the patients’ stays in the PWS unit. Anamnestic, clinical (including BMI) and social features were collected at the beginning of their time in the hospital. Genetic tests results were obtained from the caregivers or directly from laboratories and new analyses were performed when necessary (if results were not found or never done). Quantitative multiplex PCR of short fragments (QMPSF) was used to determine the size of all deletions. At the time of the study, 36 deletions were typed. Intellectual disability was assessed during a patient’s stay by an experienced clinical psychologist using the French version of the Wechsler Adult Intelligence Scale (WAIS-III); the full-scale (FSIQ), verbal (VIQ) and performance (PIQ) intelligence quotients were determined for 84 subjects. For 16 patients, assessment was not possible because of a strikingly limited capacity for understanding (5) or because of behavioral disorders (11). The data collection and analysis were conducted in compliance with the Declaration of Helsinki.

### The developmental behavior checklist for adults

The Developmental Behavior Checklist for Adults (DBC-A) is an assessment instrument completed by lay informants to assess behavioral and emotional disturbance in adults with intellectual disability. It is a recent redevelopment of an existing measure of behavior for children and adolescents with intellectual disability, the Developmental Behavior Checklist (DBC) by Einfeld and Tonge [43]. It covers 107 behavioral items rated on a three-point scale ranging from 0 (not true) to 1 (somewhat or sometimes true) to 2 (very true or often true). The DBC-A is an instrument of established reliability and validity and shows good psychometric properties. The interclass correlations for test-retest and inter-rater reliability ranges from 0.72 to 0.85 [44]. In addition to a total score, six subscales based on factor analyses can be computed. The six subscales are: disruptive, self-absorbed, communication disturbance, anxiety/antisocial, social relating and depressive. Because of the lack of a standardized scale for the DBC-A scores, we computed raw scores for the six subscales and transformed them into weighted raw scores (subscore divided by the number of items in the respective subscale). We used this procedure to compare the various subscales with each another.

DBC-A was measured for each of the patients in the sample (*n* = 100) at the end of their hospitalization (minimum 1 month, mean length 1.5 months). Nurses and carers served as informants on the basis of their observations whilst the patients were in the hospital.

In order to explore variations in behavior according to life context, we asked relatives living with patients to complete the DBC-A for the patients. A questionnaire was sent by mail to a relative once the patient had returned home. They were asked to assess the presence of a behavior in the previous six months regardless of the date of hospitalization. The response rate was 70% (*n* = 70). These scores were compared with those reported by hospital informants for the same patients.

### The hyperphagia questionnaire

The Hyperphagia Questionnaire is a 13-item instrument specifically designed to measure food-related preoccupations and problems in PWS, as well as the severity of these concerns [2]. The items measure hyperphagic symptoms reported by relatives and are rated on a five-point scale (1: not a problem to 5: severe and/or frequent problem). It gives a total score and three subscores defined by factorial analysis: behavior, drive and severity. Hyperphagia Questionnaires were sent by mail to relatives living with patients once the patient had returned home and the response rate was 75% (*n* = 75). We did not ask the hospital carers to complete the questionnaire since in the PWS unit food-related disorders were too rare to allow their assessment.

### Data analysis

Descriptive and analytical statistics were calculated using a computerized system, the SPSS-17. A parametric analysis of variance (ANOVA) test was used to determine the significance of the group differences; comparisons of genotype and gender were conducted for the DBC-A total score, subscale scores and individual items of DBC-A. Correlations between DBC-A scores and age, BMI or IQ were investigated by computing Pearson’s *r* coefficients. A Bonferroni correction was applied to adjust the correlation between DBC-A scores and BMI and between DBC-A score and PIQ. A parametric ANOVA test was used to determine the significance of the differences between mean subscale scores of the DBC-A completed by relatives and by hospital caregivers.

Non-parametric Wilcoxon tests were used to compare the distribution of scores from the Hyperphagia Questionnaire between genotype groups and between genders. Correlations between Hyperphagia Questionnaire scores and BMI, cognitive results and age were studied using Pearson’s *r* coefficients, as was the correlation between Hyperphagia Questionnaire scores and DBC scores.

The Type 1 and type 2 deletion groups were compared using the different parameters in the study using non-parametric Wilcoxon analysis.

All tests were considered to be significant if the *P* value was equal to or less than 0.05.

## Results

### DBC-A scores for the whole group, DEL and Non-DEL subgroups

Scores for the DBC-A completed by hospital caregivers were used to determine the severity and the profile of behavior disorders, the differences between genotypes and correlations with gender, age, BMI and IQ. DBC-A total and mean subscale scores are shown in Table 2 for the whole group and DEL and Non-DEL subgroups. A comparison between subgroups is also shown.

**Table 2:** DBC-A weighted raw mean scores assessed by hospital caregivers

	**Means of DBC-A scores (SD)**			***P *value between DEL and Non-DEL**^**a**^
	**Whole group**	**Deletion group**	**Non-deletion group**	
	***n *= 100**	***n *= 73**	***n *= 23**	
Total DBC-A score	0.29 (0.2)	0.26 (0.2)	0.40 (0.2)	*P* = 0.004**
Disruptive	0.56 (0.4)	0.53 (0.4)	0.66 (0.5)	NS
Self-absorbed	0.15 (0.1)	0.12 (0.1)	0.23 (0.2)	*P* = 0.004**
Communication disturbance	0.28 (0.3)	0.23 (0.2)	0.43 (0.4)	*P* = 0.001**
Anxiety/antisocial	0.17 (0.2)	0.16 (0.2)	0.20 (0.2)	NS
Social relating	0.47 (0.3)	0.41 (0.3)	0.63 (0.4)	*P* = 0.012*
Depression	0.26 (0.2)	0.25 (0.3)	0.30 (0.2)	NS

^a^*P* values from ANOVA test: * *P* ≤ 0.05, ** *P* ≤ 0.01.

*ANOVA* analysis of variance, *DBC-A* Developmental Behavior Checklist for Adults, *NS* not significant, *SD* standard deviation.

The effects of BMI and IQ on DBC-A scores were analyzed using correlation statistics controlled for genotype because this variable was found to affect BMI (*F* = 13.55, *P* = 0.000) and PIQ (*F* = 10.31, *P* = 0.002).

A partial correlation analysis performed controlling for genotype showed significant negative correlations between BMI and DBC-A total score (*r* = −.29, *P* = 0.028), self-absorbed (*r* = −.22, *P* = 0.031) and social relating (*r* = −.24, *P* = 0.021). A partial correlation analysis controlled for genotype found a negative correlation between self-absorbed and PIQ (*r* = −.30, *P* = 0.009) and between social relating and VIQ (*r* = −.32, *P* = 0.005). No significant relation was found between the DBC-A total and subscale scores and gender or age.

An item-by-item analysis of DBC-A revealed inequalities in how they corresponded to the behavioral features of PWS. Of the 107 items of the instrument, only 22 had mean scores for the whole sample exceeding 25% of the maximum (0.5 of 2). Posterior analyses were limited to these items. Table 3 presents the mean scores for these 22 items for the whole group, separated by gender and genotype, as well the results of statistical comparisons between subgroups.

**Table 3:** Mean scores for the 22 selected items from the DBC-A

		**Means of DBC-A scores (SD)**					***P *value between DEL and Non-DEL**^**a**^	***P *value between males and females**^**a**^
**Item**	**Description**	**Whole group**	**DEL**	**Non-DEL**	**Males**	**Females**		
		***n *= 100**	***n *= 73**	***n *= 23**	***n *= 44**	***n *= 56**		
1	Appears depressed, downcast or unhappy	0.55 (0.7)	0.56 (0.7)	0.52 (0.7)	0.50 (0.7)	0.59 (0.7)	NS	NS
4	Abusive, swears at others	0.66 (0.8)	**0.48** (0.7)	**1.17** (0.8)	0.64 (0.7)	0.68 (0.8)	*P* < 0.001**	NS
7	Becomes over-excited	0.64 (0.8)	0.62 (0.8)	0.83 (0.8)	0.52 (0.7)	0.73 (0.8)	NS	NS
21	Easily led into trouble by others	0.71 (0.8)	**0.60** (0.7)	**0.96** (0.8)	0.61 (0.7)	0.79 (0.8)	*P* = 0.050*	NS
28	Gorges food; will do anything to get food, for example, takes food out of garbage bins or steals food	0.73 (0.9)	0.79 (0.8)	0.52 (0.8)	0.84 (0.9)	0.64 (0.8)	NS	NS
29	Gets obsessed with an idea or activity	0.92 (0.8)	**0.78** (0.8)	**1.39** (0.7)	0.80 (0.7)	1.02 (0.9)	*P* = 0.001**	NS
34	Has temper tantrums, for example, stamps feet, slams doors	0.80 (0.7)	0.78 (0.7)	0.83 (0.8)	0.82 (0.7)	0.79 (0.7)	NS	NS
38	Impatient	0.74 (0.8)	**0.63** (0.7)	**1.09** (0.9)	0.75 (0.8)	0.73 (0.8)	*P* = 0.015*	NS
40	Increase in appetite	1.31 (0.8)	**1.40** (0.8)	**1.00** (1.0)	1.43 (0.8)	1.21 (0.9)	*P* = 0.049*	NS
41	Impulsive, acts before thinking	0.53 (0.8)	**0.40** (0.7)	**0.96** (0.9)	0.43 (0.7)	0.6 (0.8)	*P* = 0.001**	NS
42	Irritable	0.89 (0.8)	0.85 (0.7)	1.04 (0.8)	**0.86** (0.7)	**0.91** (0.8)	NS	*P* = 0.029*
43	Jealous	0.73 (0.8)	0.75 (0.8)	0.61 (0.8)	0.57 (0.8)	0.86 (0.9)	NS	NS
44	Kicks, hits or injures others	0.51 (0.7)	**0.40** (0.6)	**0.87** (0.8)	0.34 (0.5)	0.64 (0.8)	*P* = 0.004**	NS
52	Makes gloomy statements	0.58 (0.6)	0.56 (0.6)	0.74 (0.7)	0.48 (0.6)	0.66 (0.7)	NS	NS
55	Moves slowly, underactive, does little, for example, only sits and watches others	0.53 (0.8)	0.51 (0.8)	0.65 (0.9)	0.48 (0.8)	0.57 (0.8)	NS	NS
65	Prefers to do things on his/ her own; tends to be a loner	0.77 (0.9)	**0.62** (0.8)	**1.22** (0.9)	0.68 (0.8)	0.84 (0.9)	*P* = 0.003**	NS
66	Preoccupied with only one or two particular interests	0.51 (0.8)	0.47 (0.7)	0.70 (0.9)	0.50 (0.8)	0.52 (0.8)	NS	NS
75	Scratches or picks her/his skin	0.61 (0.8)	0.51 (0.8)	0.78 (0.8)	0.45 (0.8)	0.73 (0.8)	NS	NS
85	Stubborn, disobedient or uncooperative	0.63 (0.7)	**0.52** (0.7)	**0.87** (0.8)	0.64 (0.7)	0.63 (0.8)	*P* = 0.046*	NS
94	Tells lies	0.65 (0.7)	0.64 (0.7)	0.70 (0.8)	0.68 (0.7)	0.63 (0.8)	NS	NS
96	Tense, anxious, worried	0.72 (0.7)	0.68 (0.7)	0.87 (0.9)	0.66 (0.8)	0.77 (0.7)	NS	NS
102	Upset and distressed over small changes in routine or environment	0.88 (0.8)	0.81 (0.7)	1.13 (0.9)	**0.66** (0.7)	**1.05** (0.8)	NS	*P* = 0.013*

^a^*P* values from ANOVA test: * *P* ≤ 0.05, ** *P* ≤ 0.01.

*NS* non-significant, *SD* standard deviation, *DEL* Deletion group, *Non DEL* Non deletion group.

**Figure 1:**
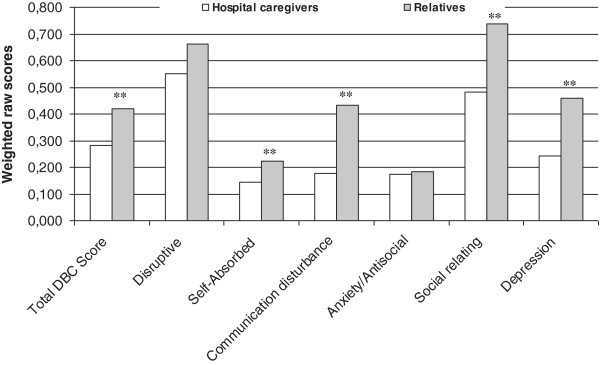
Weighted raw mean scores of DBC-A completed by relatives living with patients and hospital caregivers.

Figure 1 compares the mean total and subscale scores of the DBC-A completed by relatives and by hospital caregivers for 70 subjects. All of the measurements were higher when the informants were the relatives. Differences were significant for DBC-A total score (*t* = 3.97, *P* < 0.001), self-absorbed (*t* = 3.03, *P* = 0.003), communication disturbance (*t* = 3.84, *P* < 0.001), social relating (*t* = 3.87, *P* < 0.001) and depressive (*t* = 4.45, *P* < 0.001) but not for disruptive behavior and anxiety/antisocial.

### Hyperphagia questionnaire

The results of the Hyperphagia Questionnaire’s total score and subscales are shown in Table 4 along with the correlation analysis for age, BMI and VIQ, and the comparisons between genders and genotypes. The correlations with PIQ are not presented: there were no significant results.

**Table 4:** Results for the hyperphagia questionnaire for 75 people with PWS

**Factor**	**Mean**	**SD**	**Correlation with:**			**Differences by:**	
			**Age**	**BMI**	**VIQ**^**a**^	**Gender**	**Genotype**
			**(*n *= 75)**	**(*n *= 75)**	**(*n *= 65)**	**(M, *n *= 32, F, *n *= 43)**	**(DEL, *n *= 55, Non-DEL, *n *= 18)**
Total hyperphagic score	28.86	8.01	NS	NS	*r* = −0.258, *P* = 0.038*	NS	NS
Hyperphagic behavior	12.79	4.27	NS	NS	*r* = −0.358, *P* = 0.003**	NS	NS
Hyperphagic drive	11.50	3.31	NS	NS	NS	NS	NS
Hyperphagic severity	4.57	2.14	NS	NS	NS	NS	NS

^a^ * P ≤ 0.05, ** P ≤ 0.01.

*BMI* body mass index, *F* females, *M* males, *NS* not significant, *PWS* Prader–Willi syndrome, *VIQ* verbal IQ.

Finally, we analyzed the correlations between hyperphagia and non-food-related behavioral disturbances using the relative DBC-A scores to compare data with the same origin. The results are presented in Table 5.

**Table 5:** **Correlation between hyperphagia questionnaire and DBC-A scores**^**a**^

	**Hyperphagia questionnaire total score**	**Behavior**	**Drive**	**Severity**
Total DBC-A score	*r* = 0.411, *P* < 0.001**	*r* = 0.369, *P* = 0.002**	*r* = 0.271, *P* = 0.023*	*r* = 0.379, *P* = 0.001**
Disruptive	*r* = 0.404, *P* < 0.001**	*r* = 0.360, *P* = 0.002**	*r* = 0.307, *P* = 0.01**	*r* = 0.312, *P* = 0.009**
Self-absorbed	*r* = 0.438, *P* < 0.001**	*r* = 0.395, *P* < 0.001**	*r* = 0.313, *P* = 0.008**	*r* = 0.362, *P* = 0.002**
Communication disturbance	NS	NS	NS	*r* = 0.238 , *P* = 0.047*
Anxiety/antisocial	*r* = 0.365 , *P* = 0.002**	*r* = 0.332, *P* = 0.005**	NS	*r* = 0.348, *P* = 0.003**
Social relating	*r* = 0.255, *P* = 0.033*	NS	NS	NS
Depression	NS	NS	NS	*r* = 0.251, *P* = 0.036*

^a^ * *P* ≤ 0.05; ** *P* ≤ 0.01.

*DBC-A* Developmental Behavior Checklist for Adults, *NS* not significant.

### Differences between long and short deletions

No significant differences were found when comparing the TI and TII groups for the parameters included in this study: BMI, FSIQ, VIQ, PIQ, DBC-A scores and Hyperphagia Questionnaire scores.

## Discussion

This study describes behavioral disorders in a cohort of adults with PWS and their correlation with variables such as gender, age, BMI, IQ and life context. The size of the sample and the reliability of the observations performed by professionals with extensive experience in this syndrome could be considered as strengths. The reliability of the measurements, such as intellectual disability or BMI, was guaranteed by the homogeneity of the hospital context where they were taken. A generalization of these results to the entire adult PWS population must be made with caution. The existence of a recruitment bias cannot be totally excluded. However, our population seems to be quite representative of the PWS population since the PWS unit is not oriented to the treatment of specific symptoms and the sample presented with a wide range of the most typical features of the syndrome.

The questionnaires used do not have the norms of a reference population, which prevents a comparison with a reference population. Behavioral assessment questionnaires based on a dimensional approach and specifically oriented toward populations with an intellectual disability (such as DBC-A) could be useful for identifying behavioral problems in patients with different syndromes and for making comparisons between them. However, they may not be quite so descriptive when applied to a particular syndrome with a specific behavioral phenotype, such as PWS. In fact, in our study, only 22 items out of 107 in the DBC-A showed a clear capacity for describing the behavior of patients in the sample. In order to analyze the differences between individuals with PWS, to follow the temporal evolution of symptoms and to evaluate the effectiveness of diverse therapeutic strategies, it is necessary to create a PWS-specific behavioral assessment, such as the one for hyperphagia.

The above concerns – the need for caution in generalization and some weaknesses of the questionnaires – together with the limited number of patients with a typed deletion are the main limitations of this study.

### Behavioral profile

Our DBC-A results confirmed that behavioral disturbances have a high prevalence among subjects with PWS. The total DBC-A score when completed by relatives (0.42) was close that to reported by Sinnema *et al.* (0.47) in their recent article [14]. The behavioral profile given by the six subscale weighted raw scores was similar when the informants were caregivers or relatives living with patients, showing therefore a stable pattern. Two subscales clearly scored higher than the others: disruptive and social relating. Two others scored very low: self-absorbed and anxiety/antisocial. The remaining two, communication disturbance and depressive, scored at intermediate levels. Therefore, the most frequent behavioral disturbances in adults with PWS, those more likely to impair their social adaptation, are for externalizing aspects. The internalizing aspects were less prevalent, with anxiety-related symptoms being particularly low. This is in agreement with a previous report [19] and indicates what should be addressed by a management strategy for adults with PWS.

### Effects of genre, age, BMI and IQ on behavior

Our data are in concordance with other reports, which found little or no differences in behavior between men and women [9,14,26,45]. Women scored significantly higher than men in only two items of the DBC-A: ‘irritability’ and ‘distress over small changes in their routine or environment’.

We did not find significant changes in behavior related to age, but our sample age distribution was quite narrow; all subjects were over 18 years old and only 8% were older than 40. This may explain the lack of correlation between age and maladaptive behavior as has previously been reported [14,20,21].

The association between weight and behavioral problems is a controversial issue in the PWS literature. Our results are partially in accordance with authors who found better behavior in those with a higher degree of obesity [20], and they disagree with reports that associate high BMI scores with more behavioral disturbances [26]. In our sample, the self-absorbed and social relating scores were negatively correlated with BMI, but other dimensions that have been reported to improve with weight, such as disruptive features [22], did not do so. Any study of the relation between BMI and any symptom of PWS must be controlled for genotype because obesity is less severe in the m-UPD group than in the deletion group. This aspect could explain the lack of consistency in previous research.

Our findings are fundamentally in agreement with those of previous reports [8,14,16,26] showing that the level of cognitive impairment is not associated with severity for most of the maladaptive behaviors. Only the VIQ scores appeared to be associated with social capacity whereas PIQ scores correlated negatively with self-absorbed scores. Further research is needed in order to better understand the role of specific cognitive processes in emotional and behavioral disturbances.

### Life context and behavior

We compared the DBC-A scores when completed by relatives with those completed by hospital caregivers as a way to analyze the influence of life context on behavior. Even if the times of the observations varied, this comparison is possible because DBC-A assesses behaviors that are characteristic of the global functioning of the PWS patients, as structural issues independent of acute events. The total DBC-A score was significantly lower in the hospital set.

The fact that relatives scored higher than the hospital caregivers in most (four) of the DBC-A subscales could be due to two factors. The first factor is that the hospital caregivers could have underscored because they are used to coping with behavioral challenges and their reference or baseline could be higher than those of the relatives. However, interestingly, not all subscales were affected by this difference. The other factor is the beneficial effect of being in the hospital. As the patients often refer themselves, the security and confidence that a structured environment creates must allow them to feel freer from their compulsive concerns and available for more adaptive activities, for example, interpersonal relationships. The fact that the subscales disruptive and anxiety/antisocial were rated similarly by both relatives and hospital caregivers indicates what aspects are more context dependent in PWS behavior and what are more structural: disruptive features are frequent and signs of anxiety are rare in all circumstances. However, the communication, social relating and depressive symptoms can improve dramatically in an environment that keeps food-related concerns under control and facilitates interactions with other PWS patients. This must be taken into account when assessing emotional distress and in developing care strategies for people with PWS.

### Hyperphagic behavior

Hyperphagia is the most salient and constant behavior disorder in PWS and all approaches to controlling it have proven to be ineffective. The Hyperphagia Questionnaire scores were very close to those reported by the authors of a study on a PWS population in the USA [2]. This reinforces the validity of this instrument for assessing eating behavior for PWS patients across different cultural environments. Our results agree with those of Dykens *et al*. [2] concerning the lack of significant relations between hyperphagia scores and genetic status, but in contrast to their report we did not find any correlations when the hyperphagia scores were compared with BMI or age. A reason for this could be that our sample was limited to an adult population. Another difference concerns the effect of cognitive impairment. In contrast with their results, we found a strong negative correlation between verbal ability (VIQ) and hyperphagia behavior.

The relationships between the Hyperphagia Questionnaire scores and the other maladaptive behaviors assessed with the DBC-A raise to a reflexion on the interplay between hyperphagia and other behavioral and emotional problems in PWS. In contrast to the results reported by Dykens *et al*. [2], we found that non-food-related behavior scores correlated positively with all of the subscales of the Hyperphagia Questionnaire, perhaps because the assessment instruments were not the same (they used the Aberrant Behavior Checklist). Therefore, we conclude that food-related and unrelated behavior disturbances are closely associated, although the cause-effect relationship is not clear. However, when food-related concerns were minimized because of institutional control during hospitalization, we found a striking improvement in some aspects of behavior (depressive features), whereas disruptive behavior remained similar. This supports the idea that even if some emotional and behavioral symptoms are related to the intensity of the hyperphagia challenge, other symptoms, such as disruptive features, are not related and so they must be considered as an independent expression of the behavioral phenotype in PWS. Therefore, different physio-pathological mechanisms should be considered.

### Differences between genotypes

This study found significant differences between the deletion (the DEL group) and m-UPD (the Non-DEL group) genotypes in several behavioral issues. Enriching the group of confirmed m-UPD diagnoses with the 13 patients who had an abnormal methylation profile and negative results for the deletion diagnosis could be considered as a risk factor in the interpretation of these differences. However, it must be considered that imprinting defects are rare (2% to 5%) and, moreover, epigenetic mutations of the imprinting center lead to a maternal DNA-methylation pattern on both chromosomes, which is genetically similar to m-UPD.

Concerning the behavioral profile (DBC-A subscale scores), the difference was quantitative rather than qualitative. Thus, both had the same pattern but the Non-DEL group scored higher than the DEL group in all of the subscales. This difference was significant for self-absorbed, communication disturbance and social relating. When compared item by item, significant differences were found in nine items and the Non-DEL group always scored higher than the DEL group, except for ‘increase in appetite’. Impulsive, obsessive or aggressive features were more frequent in the Non-DEL group. These data agree with a recent report [14] and differ from previous work that found greater levels of maladaptive behavior in the deletion genotype compared to the m-UPD genotype [16,22]. A reason for these discrepancies could be the age of our adult sample. Behavioral problems could improve from childhood to adulthood particularly in deletion cases. In contrast, the more frequent presence in disomy cases of psychosis [33,39,46] and autistic-like symptoms [47] would explain the higher prevalence of behavioral disorders in adults. The DBC-A is surely more sensitive for psychopathological features than other instruments measuring the presence of maladaptive behaviors independently of emotional disorders. This would explain our agreement with the report of Sinnema *et al*., who also employed the DBC-A.

With respect to behavioral differences across PWS paternal deletion subtypes, our results are in accordance with those of Dykens and Roof [29], who did not find compelling differences between type I and type II deletions, and disagree with previous reports [22,40,41]. However, the size of our sample is small and our results should be considered as preliminary findings. Further research is needed to clarify the discrepancies.

Differences between the genetic subtypes of PWS have important implications for understanding the genetic basis of this syndrome. It has been hypothesized that phenotypic differences between the deletion and m-UPD genotypes are related to molecular differences, such as the haploid insufficiency of non-imprinted genes in deletion cases or, in UPD cases, the overexpression of genes that are normally only expressed on the maternally derived part of chromosome 15q11-13, such as UBE3A and ATP10C [48]. However, recent reports of atypical deletions in the PWS chromosomal region have introduced new perspectives concerning the molecular mechanisms implicated in PWS [49,50].

## Conclusions

This study, in agreement with previous reports on psychiatric and cognitive differences [28] between the deletion and m-UPD subtypes, reinforces the idea that there are two different phenotypes in PWS with respect to cognitive, behavioral and psychiatric profiles.

Future research is necessary to give further knowledge of the neural and neuropsychological mechanisms underpinning these differences. Furthermore, these differences should be taken into account when developing therapeutic and management strategies to help patients with PWS and their families.

The increased prevalence of behavioral problems in adults with the m-UPD genotype contradicts some of the literature for PWS, except for the recent report of Sinnema *et al*. [14], who also studied adults with PWS and used the same questionnaire, DBC-A, which may be more sensitive to psychopathology in adults with PWS.

The current study highlights the importance of following individuals with PWS throughout their lives to ensure that effective targeted treatments are implemented at critical times.

## Abbreviations

ANOVA: Analysis of variance; BMI: Body mass index; DBC-A: Developmental behavior checklist for adults; FSIQ: Full-scale IQ; IQ: Intelligence quotient; m-UPD: Maternal uniparental disomy; PIQ: Performance IQ; PWS: Prader–Willi syndrome; TI: Type I deletion; TII: Type II deletion; VIQ: Verbal IQ.

## Competing interests

The authors declare that they have no competing interests.

## Authors’ contributions

JJ managed this work and was a major contributor in writing the manuscript. VL and PC conducted the tests. DT and MT are the coordinators at the center. All authors read and approved the final manuscript.
